# En bloc versus conventional resection of primary bladder tumor: a systematic review and meta-analysis

**DOI:** 10.1016/j.clinsp.2026.101065

**Published:** 2026-07-23

**Authors:** Pietro Delgado Rezende, Adriana Sayuri Hashimoto, Henrique Lepine, José Pedro Cassemiro Micheleto, Mauricio Dener Cordeiro, José Maurício Mota, William Carlos Nahas, Mohamed Elkoushy, Walid Shahrour, Walid Shabana, Marian Severin Wettstein, Leopoldo Alves Ribeiro-Filho, Caio Vinicius Suartz

**Affiliations:** a Division of Urology, Instituto do Câncer do Estado de São Paulo (ICESP), Universidade de São Paulo (USP), São Paulo, SP, Brazil; b Department of Urology, Thunder Bay Regional Health Sciences Centre and Northern Ontario School of Medicine University, Thunder Bay, ON, Canada

**Keywords:** “Bladder transurethral resection”, “Bladder cancer, Non-muscle-invasive”, “Clinical trials, Randomized”, “Systematic review”

## Abstract

**Objective:**

To provide an updated view comparing the perioperative, functional characteristics and oncological outcomes in patients submitted to cTURBT and ERBT for the diagnosis and treatment of NMIBC.

**Methods:**

A search of major databases, including Medline, Embase, Lilacs, Scopus, Web of Science, NIH, Clinical Trials, and EU Clinical Trials Register, was conducted up to January 2025, adhering to PRISMA guidelines. Only Randomized Controlled Trials (RCTs) were included. The primary endpoints assessed were perioperative complications and oncological outcomes.

**Results:**

This study encompasses 16 studies including 2.654 patients. Surgery duration showed a longer time of operation in the ERBT group (MD = 3.09, 95% CI 0.15‒6.03). Also, less time of catheterisation (MD = -0.54, 95% CI -0.97 ‒ -0.11) and shorter length of stay in the ERBT group (MD = -0.83, 95% CI -1.41 ‒ -0.25). The total number of perioperative complications was lower in the ERBT group, although no statistically significant difference was shown between groups. Lastly, the ERBT group was associated with less chance of triggering the obturator reflex (OR = 5.44, 95% CI 1.61‒18.34). Oncological outcomes showed no significant difference between groups.

**Conclusion:**

ERBT and cTURBT showed no difference in short-term oncological outcomes. Although ERBT did not reduce overall complication rates, it demonstrated a benefit in reducing the occurrence of the obturator reflex and shorter time of hospitalization. Further randomized controlled trials with long-term follow-up and a broad spectrum of tumor sizes are necessary. More randomized controlled trials are needed, focusing on long-term follow-up and a wider range of tumor sizes.

## Highlights

• ERBT reduces the risk of obturator reflex but does not significantly reduce overall complication rates compared to cTURBT.
• ERBT needs fewer days of hospitalization and of indwelling catheter.
• Oncological outcomes showed no difference comparing ERBT or cTUBRT.
• Longer follow-up is needed to compare oncological outcomes.

## Introduction

Bladder Cancer (BC) is a major global health issue, ranking as the tenth most diagnosed cancer and a leading cause of morbidity and mortality worldwide.^1^ In men, it is the sixth most common cancer.^2^ According to GLOBOCAN, 573,000 new BC cases and 213,000 related deaths occurred in 2020.^1^^,^^3^ Non-Muscle-Invasive Bladder Cancer (NMIBC) accounts for about 75% of cases and includes a heterogeneous group of patients with variable risks of recurrence and progression to Muscle-Invasive Disease (MIBC).^4^

Transurethral Resection of Bladder Tumor (TURBT) is the standard of care for diagnostic and therapeutic approach in NMIBC patients.^5^ However, conventional TURBT (cTURBT) has been criticized for violating the oncologic “no-touch” principle, causing thermal damage, tissue fragmentation, and cauterization artefacts that hinder accurate staging.^6^ Moreover, absence of detrusor muscle in 15.3%–51% of specimens^7^ increases the risk of residual disease, early recurrence, and tumor under-staging.^8^ En bloc Resection of Bladder Tumor (ERBT) was developed in an attempt to improve specimen integrity and overcome these limitations^9^, ^10^, ^11^, ^12^ Evidence from retrospective series and Randomized Controlled Trials (RCTs) suggests ERBT provides more accurate staging, fewer complications, and lower recurrence rates than Cturbt.^12^

This study presents a systematic review and meta-analysis of only RCTs comparing perioperative, functional, and oncological outcomes between cTURBT and ERBT.

## Material and methods

The review followed the PRISMA guideline and was prospectively registered in the PROSPERO database (CRD42024590589) in September 2024. An initial search was completed in October 2024, with an updated search conducted in January 2025.

A research question was developed using the PICOS framework:^13^ Is there a difference in oncological outcomes between the en bloc and conventional approaches to bladder TURBT in patients with NMIBC?

The search strategy was developed to identify randomized studies comparing en bloc resection of bladder tumor with conventional transurethral resection. Controlled vocabulary and free-text terms were combined, including “Bladder Transurethral Resection”, “transurethral resection of bladder tumor”, “TURBT”, “non-muscle-invasive bladder cancer”, “NMIBC”, and “randomized clinical trial”, The literature search was performed through January 2025 in MEDLINE (Ovid), Embase (Ovid), LILACS, Scopus, and Web of Science. Trial registries, including ClinicalTrials.gov, ReBEC, and the EU Clinical Trials Register, were also searched. Reference lists of the included studies were manually screened for additional eligible articles.

Only randomized clinical trials published in English were eligible for inclusion. The eligibility criteria were established according to the PICOS framework: Population, adults aged 18-years or older with urothelial bladder tumors; Intervention, en bloc resection of bladder tumor; Comparator, conventional transurethral resection of bladder tumor; Outcomes, with oncological outcomes as the primary endpoint and perioperative complications as secondary endpoints; and Study design, randomized controlled trials without restriction on publication date.

### Data extraction and endpoints

The data were entered into an Excel® sheet by 2 authors and cross-checked by a third reviewer. Continuous variables were recorded as mean ± standard deviation; medians and interquartile ranges were converted when necessary.^14^ The authors collected the following variables: number of patients, age, mean follow-up, energy source, instillation treatment, clinical-pathological staging, tumor size and location, number of lesions, pathological stage, surgery duration, hospital stay, recurrence-free survival, 12-month recurrence, obturator reflex, bladder perforation, catheter duration, residual tumor at re-TURBT, postoperative bladder irrigation time, detrusor rate detection, perioperative estimated blood loss, and perioperative complications (Clavien-Dindo classification) .^15^

Minor complications were grades I–II, while major was grades III–V. Obturator reflex, residual tumor, and detrusor detection were recorded as dichotomous variables. Perioperative and oncological outcomes were recorded as continuous variables.

Complications were categorized as minor (grades I–II) or major (grades III–V). Obturator reflex, residual tumor, and detrusor detection were documented as binary (dichotomous) outcomes. Both perioperative and oncological outcomes were recorded as continuous variables.

### Quality assessment and risk of bias

The risk of bias was analyzed with ROB-2,^16^ a tool developed by the Cochrane Collaboration for assessing the risk of bias in randomized trials. Studies were rated as having a high, low, or some concerns risk of bias across five domains: selection, performance, detection, attrition, and reporting biases. Publication bias was examined using funnel-plot analysis of point estimates according to study weights and Egger's regression test.

### Statistical analysis

**Fig. 1:**
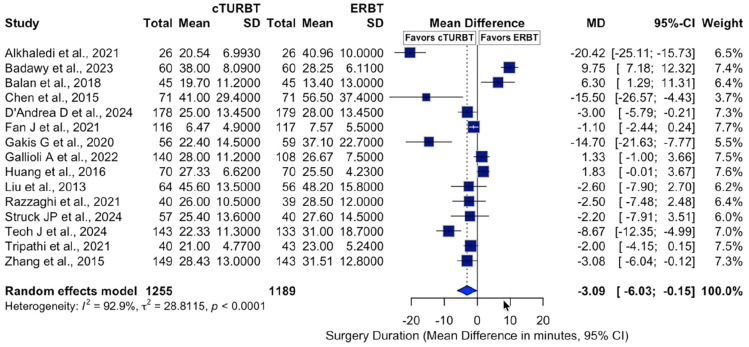
Meta-analysis of the surgery duration.

This systematic review with meta-analysis was performed in accordance with the Cochrane Collaboration and the Preferred Reporting Items for Systematic Reviews and Meta-Analysis (PRISMA) statement guidelines^17^ utilizing the Review Manager 5.4 (Cochrane Centre, The Cochrane Collaboration, Denmark), as viewed in Fig. 1. Odds-Ratios (OR) with 95% Confidence Intervals were used to compare treatment effects for categorical endpoints. Cochrane *Q*-test and I^2^ statistics were used to assess for heterogeneity; p-values inferior to 0.10 and I^2^ > 25% were considered significant for heterogeneity. The authors used a fixed-effect model for outcomes with low heterogeneity (I^2^ < 25%). Otherwise, a DerSimonian and Laird random-effects model was used.

## Results

**Fig. 2:**
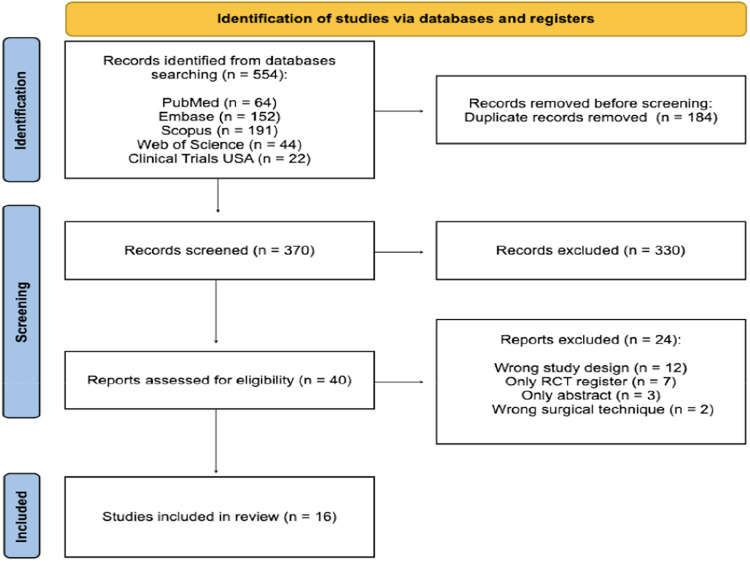
PRISMA flow diagram of the study.

The literature search retrieved 554 studies. After the automatic exclusion of duplicates, 370 studies remained. Following the title and abstract reading, 330 articles were excluded for being incompatible with the study's aim. Forty articles were selected for full-text reading. 24 reports were excluded and only 16 were selected and included for statistical analysis. Fig. 2 shows the PRISMA flowchart of the literature search.

In relation to the bias analysis, Struck et al.^18^ and David D’Andrea were deemed to have some concerns in D1 due to baseline differences between intervention groups, suggesting a problem with the randomization process ‒ the ERBT group has more males and smokers with more pack-years; Balan et al.^19^ did not include data about the participants excluded from analysis and Gakis et al. did not include information about the lost to follow up participants, so there is not evidence that the result was not biased by missing outcome data; In study of Zhang et al.,^20^ Badawy and Chen et al. there are not information’s about participants, carers and people delivering the interventions aware of participants' assigned intervention during the trial awareness of their assigned intervention during the trial, the Supplementary Figure 1 shows the study of risk of bias. The visual inspection of the funnel plot showed a slight asymmetry, suggesting possible small-study effects or publication bias, as shown in the Supplementary Figure 2. However, Egger’s regression test did not reach statistical significance (*p* = 0.1686), indicating that while some asymmetry is present, it may not substantially invalidate the pooled estimates.

All studies were RCTs comparing patients undergoing ERBT with cTURBT. A total of 2654 patients were included, with 1101 undergoing ERBT and 1553 undergoing cTURBT.

The studies were conducted in three continents: eight in Asia, five in Europe, and three in Africa. The study reporting periods vary from 2006 to 2022. In all studies, the majority of participants were male. Table 1 presents the baseline characteristics of the included studies.

**Table 1:** Baseline characteristics of included studies.

Study	Country	Study period	Study design	Number of patients (n)		Age (yr), mean (SD)		Gender, n (%)				Follow-up (mo)
				cTURBT	ERBT	cTURBT	ERBT	cTURBT		ERBT		
								Male	Female	Male	Female	
Struck, 2025.^18^	Germany; Austria; Czech Republic; Italy	2019–2022	Randomized	40	57	69.4 (10.37)	70.2 (11.09)	32 (80.0)	8 (20.0)	38 (66.7)	18 (31.6)	24
Yuen-Chun Teoh, 2024.^21^	China	2017–2020	Randomized	133	143	69 (62–79)	70 (73–69)	110 (83)	23 (17)	108 (76)	35 (24)	12
D'Andrea, 2023.^22^	Austria	2019–2022	Randomized	179	178	70 (60–76)	66 (56–73)	140 (78)	39 (22)	133 (75)	45 (25)	24
Badawy, 2023.^23^	Egypt	2019–2021	Randomized	60	60	61.83±7.67	64.53±8.48	57 (95)	3 (5)	55 (91.7)	5 (8.3)	12
Gallioli, 2022.^24^	Spain	2018–2021	Randomized	108	140	73 (65–79)	72 (61–80)	91 (84)	17 (16)	109 (78)	31 (22)	15
Tripathi, 2021.^25^	India	2019–2021	Randomized	43	40	56.12 ± 12.11	55.62 ± 12.75	30 (70)	13 (30)	32 (80)	8 (20)	12
Razzaghi, 2021.^26^	Iran	2017–2019	Randomized	39	40	68.2 ± 9.8	65.8 ± 10.8	35 (89.7)	4 (10.3)	38 (95)	2 (5)	18
Alkhaledi, 2021.^27^	Egypt	2017–2018	Randomized	26	26	x	x	x	x	x	x	1,5
Fan, 2021.^28^	China	2014–2018	Randomized	117	116	57 (49–64)	60 (52–67)	87 (74)	30 (26)	96 (83.2)	20 (16.8)	48
Hashem, 2021.^29^	Egypt	2015–2018	Randomized	50	50	61.1 (11.3)	60.4 (11.9)	39 (78)	11 (22)	37(74)	13 (26)	20
Gakis, 2020.^30^	Germany	2012–2015	Randomized	59	56	70.2 (12.4)	66.8 (11.1)	47 (79.7%)	12 (20.3%)	45 (80.4%)	11 (19.6%)	12
Balan, 2018.^19^	Romania	2014–2018	Randomized	45	45	66.1	64.7	x	x	x	x	12
Huang, 2016.^31^	China	2009–2013	Randomized	70	Thulium 70	57.85 (4.99)	Thulium 58.31 (6.13)	48 (68.6)	22 (31.4)	Thulium 50 (71.4)	Thulium 20 (28.6)	24
					Holmium 70		Holmium 59.97 (5.75)			Holmium 45 (64.3)	Holmium 25 (35.7)	
Zhang, 2015.^20^	China	2006–2010	Randomized	143	149	<60 35 (24.5)	<60 42 (28.2)	79 (55.2)	64 (44.8)	70 (46.9)	79 (53.1)	36
						61–70 63 (44.1)	61–70 57 (38.3)					
						71‒80 26 (18.2) > 80 19 (13.3)	71‒80 30 (20.1) > 80 20 (13.4)					
Chen, 2015.^32^	China	2008–2011	Randomized	71	71	62 (20–89)	63 (25–90)	51 (71.8)	20 (28.1)	54 (76.0)	17 (24.0)	18
Liu, 2013.^33^	China	2006–2008	Randomized	56	64	66.3 (9.8)	67.1 (8.3)	40 (71.4)	16 (28.6)	46 (71.9)	18 (28.1)	36

Fourteen studies reported tumor staging, with pTa being the most common, followed by pT1. Two studies did not provide staging data. Tumor localization was described in 15 studies, tumor diameter in 14, and the number of lesions in 11. The rate of complete resection was reported in eight studies, and detrusor muscle detection in 10. Detailed information is presented in Supplementary Table 1.

Energy sources varied across studies: four used Thulium laser, three Holmium laser, one both Thulium and Holmium, two bipolar, one KTP, one green-light, one HybridKnife®, and three multiple sources (bipolar, monopolar, and laser). Additionally, three studies used enhanced tumor visualization techniques.

Twelve studies reported intravesical instillation therapies, including BCG, epirubicin, mitomycin, or doxorubicin. Further details are shown in Supplementary Table 2.

Surgery duration was reported in 15 studies, showing a significant difference between groups and a longer time of operation in the ERBT group (MD = 3.09, 95% CI 0.15‒6.03). Given the high heterogeneity (I^2^ = 92.9%, *p* < 0.0001), these pooled estimates should be interpreted with caution (Fig. 1).

**Fig. 3:**
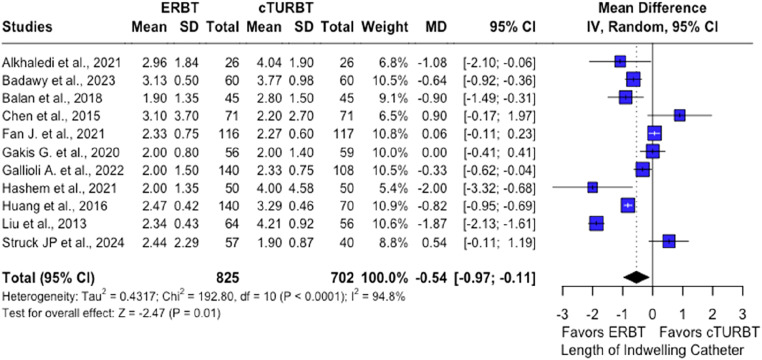
Meta-analysis of the length of the indwelling catheter.

The time of bladder irrigation after surgery was compared in six studies without statistical significance (MD = 1.39, 95% CI −1.60‒4.38), while the length of indwelling catheter was measured in eleven studies, with lower time of catheterisation in the ERBT group (MD = −0.54, 95% CI −0.97 ‒ −0.11), as shown in Fig. 3.

**Fig. 4:**
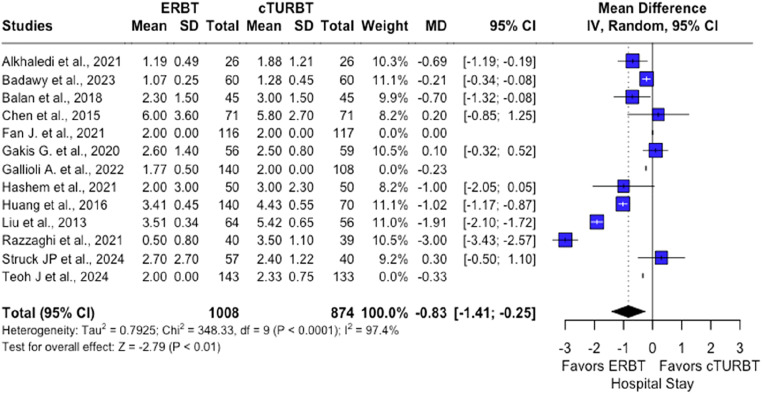
Meta-analysis of length of hospital stay.

The length of hospital stay was obtained from ten studies. The meta-analysis demonstrated a significant difference between groups, with shorter duration of hospitalization in the ERBT group (MD = −0.83, 95% CI −1.41 ‒ −0.25), presenting a high heterogeneity (I^2^ = 97.4%, *p* < 0.0001). Due to the high heterogeneity, this finding should be interpreted with caution. These findings are shown in Fig. 4.

Perioperative complications were reported in seven articles, and complications were classified according to the Clavien-Dindo system [15] as major or minor in five and seven studies, respectively. The total perioperative complications showed fewer events in the ERBT group than cTURBT (OR = 2.39, 95% CI 0.99‒5.81). No statistically significant difference was shown in minor complications (OR = 1.78, 95% CI 0.78‒4.07) or major complications (OR = 0.77, 95% CI 0.18‒3.19). Intraoperative complications were reported separately in seven studies, and showed no statistically significant difference (OR = 3.90, 95% CI 0.74‒20.70).

**Fig. 5:**
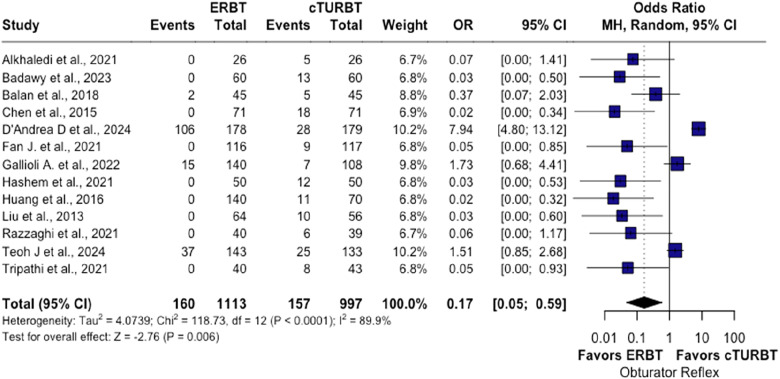
Meta-analysis of obturator reflex.

Thirteen studies reported the association of the resection technique with the presence of obturator reflex. The forest plot, presented in Fig. 5, revealed that the ERBT group was statistically significantly associated with a lower chance of triggering the obturator reflex (OR = 0.17, 95% CI 0.05‒0.59), with a high heterogeneity (I^2^ = 89.9%, *p* < 0.0001).

The rate of bladder perforation was reported in 13 studies, with no statistically significant difference between the ERBT and cTUBRT groups (OR = 1.99, 95% CI 0.76–5.19).

**Fig. 6:**
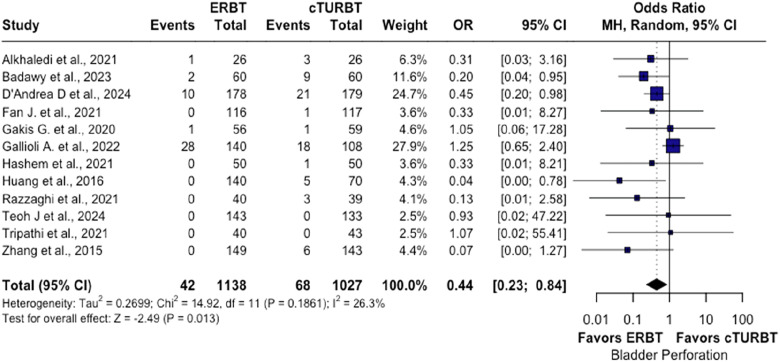
Meta-analysis of bladder perforation only with studies presenting patients with bladder tumour size ≤ 3 cm.

Therefore, a subgroup analysis restricted to studies including bladder tumors ≤ 3 cm ‒ excluding the EBRUC II trial by Struck et al. (2024), which included tumors > 3 cm and did not report obturator reflex outcomes ‒ demonstrated a statistically significant reduction in bladder perforation rates in the ERBT group compared with the cTURBT group (OR = 0.44, 95% CI 0.23–0.84) (Fig. 6).

**Fig. 7:**
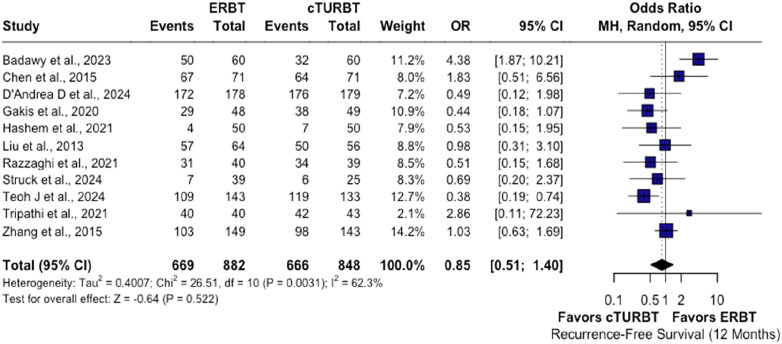
Meta-analysis of recurrence free-survival in 12-months.

Ten studies evaluated the association between resection technique and detrusor muscle detection, and showed no significant difference between the ERBT and cTURBT groups (OR = 0.64, 95% CI 0.37–1.11), with high heterogeneity (I^2^ = 75.7%, *p* < 0.0001). Residual tumor detection at re-TURBT was reported in four studies, also showing no significant difference between groups (OR = 1.16, 95% CI 0.54–2.50), with low heterogeneity (I^2^ = 0.0%, *p* = 0.93). Recurrence-free survival at 12-months was reported in eleven studies (Fig. 7), revealing no significant difference between ERBT and cTURBT (OR = 0.85, 95% CI 0.51–1.40). Similarly, twelve-month recurrence, reported in nine studies, showed no statistical difference (OR = 1.42, 95% CI 0.97–2.06).

## Discussion

This updated systematic review and meta-analysis included 16 randomized controlled trials and 2654 patients with NMIBC. The present analysis demonstrated that ERBT and cTURBT yielded comparable short-term oncological outcomes. Specifically, no significant differences were observed in recurrence-free survival, 12-month recurrence, residual tumor at re-TURBT, or detrusor muscle detection. However, ERBT was associated with some perioperative advantages, including a lower risk of obturator reflex and bladder perforation, shorter catheterization time, and reduced length of hospital stay, although operative time was slightly longer, and overall complication rates were not significantly different between approaches.

In the EBRUC II trial, [18] which imposed no restrictions on tumor number, location, or size, en bloc extraction was feasible in 83.6% of ERBT cases, whereas 16.4% required intravesical fragmentation before specimen retrieval (*p* < 0.001). Two patients (3.5%) required conversion to cTURBT. Similarly, Teoh et al.^34^ reported an 88% technical success rate for ERBT, with 12% of cases requiring conversion to cTURBT because of technical challenges, particularly for dome, solid, or sessile tumors. Two additional patients (1.4%) underwent modified ERBT. Notably, all analyses followed a modified intention-to-treat principle, and patients initially assigned to ERBT who required conversion remained in the ERBT group. For tumors too large for complete en bloc removal, a modified approach was adopted, consisting of piecemeal resection of the exophytic component followed by en bloc resection of the tumor base. Although a significant difference in 1-year recurrence was observed, the study was not powered for this secondary endpoint. Gallioli et al.^24^ also reported six conversions (4.3%) from ERBT to cTURBT, five due to anterior wall lesions and one due to a tumor near the ureteral meatus.

The tumor size feasible for retrieval en-bloc is limited by the currently available endoscopic equipment and it has been shown that technical success declines with tumours larger than three centimeters, with technical success rates of 84.3% for bladder tumor size of ≤ 3 cm decreasing to 29.6% for tumor size of > 3 cm.^34^ Bladder lesions smaller than the width of the wire loop can be resected in one piece, so tumors of < 0.5 cm^16^^,^^24^ and even < 1.0 cm^19^, ^22^, ^27^, ^30^ were excluded from inclusion criteria of several studies to prevent en bloc resection during cTURBT.

In the EBRUC II trial, there were no restrictions on the maximum tumor diameter, with intravesical fragmentation of the lesion before extraction if necessary, showing an increasing rate of tumours being fractionated intravesically starting at ∼3 cm with an increase at 3.5 cm. Similar difficulties were reported by Zhang et al.;^20^ in their study, when tumor size exceeded 3 cm, it was necessary to incise the lesion and the bladder wall base longitudinally or crosswise, which allowed the tumor to be divided into two or more parts for extraction. Other studies that did not place a limit on the maximum diameter obtained lesions smaller than 3 cm^33^ or did not report this information.^32^, ^35^, ^36^ Most studies include lesions of up to 3 cm in their inclusion or exclusion criteria, which limits the information available for larger tumors.

Overall, these findings suggest that ERBT is a safe technique and may reduce perforation rates in tumors measuring up to 3 cm. However, as reported across studies, technical difficulties become more frequent with larger lesions. A structured learning curve prioritizing tumors ≤3 cm in favorable positions may facilitate wider adoption of the technique. In addition, lasers with improved coagulation properties and more favorable energy dispersion may help expand the size threshold for safe and effective en bloc resection.

Across the included trials, ERBT was generally associated with lower rates of obturator reflex. This advantage likely reflects, at least in part, the predominant use of laser energy in the en bloc arm. In contrast to monopolar or bipolar resection, laser-based approaches minimize direct electrical stimulation of the obturator nerve and may therefore reduce reflex contraction.^37^, ^38^ A notable exception was the study by Gallioli et al.,^24^ in which obturator reflex occurred in 15 patients (11%) in the ERBT group and in 7 (6.5%) in the cTURBT group (*p* = 0.2). In that trial, higher rates were observed when bipolar energy was used in both arms, with events occurring in 4 patients (7.8%) in the cTURBT group and 10 patients (22%) in the ERBT group. No further analysis was provided to explain this discrepancy. However, the high proportion of lateral wall tumors in that cohort, 70 cases (48%) in the cTURBT arm and 107 (55%) in the ERBT arm, may have increased the baseline risk of obturator reflex irrespective of resection strategy. These findings suggest that the lower incidence of obturator reflex observed with ERBT should be interpreted cautiously, as it may be related more to the energy source than to the en bloc principle itself.

ERBT was performed in the great part of the studies using Thulium or Holmium lasers, whereas cTURBT relied on conventional electrical energy. Consequently, the observed benefits, such as the significant reduction in the obturator reflex and bladder perforation and potential improvements in hemostasis, may be attributable to the use of laser technology rather than the en bloc technique per se. Until large-scale trials compare laser-ERBT against laser-cTURBT, the observed benefits of ERBT may be attributable to the energy source rather than to the en bloc technique itself.

Laser settings were not reported consistently across studies, which precluded a fair comparison between techniques, as pulse mode, energy output, and frequency may influence the ease of resection, operative efficiency, and potentially clinical outcomes. This lack of procedural granularity, together with the absence of randomization and head-to-head comparisons across the energy sources used for ERBT, limits the interpretability of the available evidence. Future trials should standardize laser parameters and directly compare different en bloc platforms to clarify whether the observed advantages are driven by the energy source itself or reflect the intrinsic benefits of the en bloc resection principle. Such studies are essential to disentangle the effect of technology from that of surgical technique and to strengthen the internal validity of this field.

The rate of bladder perforation differed significantly between ERBT and cTURBT. This finding should be interpreted with caution, as it may be partly explained by heterogeneity and potential underdiagnosis related to inconsistent definitions and assessment methods across the included studies. In several studies, bladder perforation was not reported,^21^, ^26^, ^28^, ^31^ was based solely on clinical suspicion,^23^^,^^29^ or was assessed only by intraoperative visual inspection,^20^, ^22^, ^24^, ^25^, ^27^, ^30^ whereas only one study confirmed perforation using cystography.^39^ Moreover, most included studies were not specifically designed to evaluate bladder perforation, as this outcome was typically reported as a secondary endpoint. Therefore, well-designed studies with standardized definitions and adequate statistical power are needed to more accurately assess bladder perforation outcomes.

Surgery duration was modestly longer in the ERBT group, with a mean difference of approximately 3 min. However, seven studies reported no statistically significant difference in operative time between techniques.^18^, ^24^, ^25^, ^26^, ^28^, ^40^, ^41^ In the study by Fan et al.,^28^ ERBT was associated with a longer resection time, whereas total operative time did not differ between groups. This finding may be explained by the additional time required for hemostatic electrocoagulation during cTURBT, a step that may be reduced when high-power laser platforms with superior coagulative properties are used. It should also be acknowledged that, in en bloc techniques, large tumors that cannot be extracted intact through the resectoscope often require intravesical fragmentation or morcellation before retrieval, which may further contribute to longer operative times.

In Fan et al.,^28^ the volume of estimated blood loss was significantly lower in the ERBT group than in the cTURBT group with a median Hb decrease of 7.0 (4.0‒13.0) g/L vs. 11.0 (5.0‒18.8) g/L, *p* = 0.012, respectively. This result was highlighted as one of the advantages of using a laser in en bloc resection, which has excellent hemostatic properties. This point was also highlighted in other studies^23^, ^25^, ^42^ with Thulium laser, which may have excellent hemostasis due to continuous energy output, which makes a thin layer of eschar on the surface of the wound with clear cutting depth identification. The duration of indwelling catheterization was significantly shorter in the ERBT group, likely reflecting a more favorable perioperative recovery, with less bleeding and fewer events requiring prolonged bladder drainage. This may be partly explained by the ability of laser platforms to simultaneously cut, vaporize, and coagulate tissue, thereby improving hemostasis. Consistent with this finding, length of hospital stay was also significantly shorter after ERBT. In routine practice, catheter removal is generally performed after bladder irrigation has been discontinued and hematuria has resolved. Although postoperative irrigation time, assessed in six studies, did not differ significantly between groups, and no difference was observed in two individual trials,^18^^,^^34^ catheter duration was evaluated in a larger number of studies and consistently favored ERBT. From a health systems perspective, shorter catheterization and reduced hospitalization may also translate into lower resource utilization and lower overall costs.

Regarding oncological outcomes and the quality of muscularis propria sampling, despite the theoretical oncological advantage of ERBT in providing higher R0 resection rates and better specimen quality, as highlighted in studies such as EBRUC II,^18^ the meta-analysis did not demonstrate a significant benefit in recurrence-free survival. This discrepancy suggests that, although ERBT may yield a more intact and pathologically informative specimen, this advantage does not necessarily translate into improved short-term oncological control.^43^, ^44^, ^45^ In other words, superior specimen quality may enhance histopathological assessment without materially changing the biological course of NMIBC or the risk of microscopic tumor cell seeding during resection.

As evidenced in other studies,^26^, ^32^, ^41^, ^46^ the use of ERBT or cTUBRT did not impact on the oncological outcomes. Even though the detrusor muscle detection in sampling rates and the recurrence in 12-months tend to favor the ERBT group, the study reveals no significant statistical difference. Also, the detection of tumor in residual RE-TURBT and the recurrence-free survival showed no statistically significant difference. This suggests that when performed by experienced surgeons, both techniques can yield comparable specimen quality for pathological evaluation.^45^, ^47^, ^48^

Furthermore, the clinical adoption of ERBT faces practical hurdles. The incorporation of laser technology (such as Thulium or Holmium) involves a high economic burden for healthcare systems compared to the widely available and low-cost bipolar energy used in cTURBT. Additionally, ERBT is associated with a steeper learning curve, which may explain the higher operative times observed in the present results. This reinforces the idea that experienced surgeons can effectively resect NMIBC using either technique while maintaining the quality of oncological outcomes, suggesting that surgical expertise may be more decisive than the specific resection method employed.

Despite these inherent limitations in the quality and design of the available evidence, this review included only randomized controlled trials, encompassing 16 studies and 2654 patients with NMIBC, and provides clinically relevant findings. The main limitations of this meta-analysis are the marked heterogeneity across studies, particularly regarding the energy sources used in both en bloc resection and conventional TURBT, as well as the variability in device settings, which were often poorly specified or not reported at all. Another important limitation is the lack of patient-centered outcomes, such as quality of life and procedure-related costs, in most included analyses. In addition, the absence of standardized enhanced visualization methods, including filters such as narrow-band imaging or blue-light cystoscopy, further weakens the comparability of the included studies. Further well-designed randomized controlled trials with comprehensive and detailed reporting are needed in the near future, including studies directly comparing different en bloc resection platforms and using new biomolecular markers, such as cell-free tumor DNA (ctDNA), to better predict prognosis and therapy efficacy.^49^, ^50^

## Conclusion

In this meta-analysis of randomized trials involving patients with NMIBC, en bloc resection and conventional transurethral resection were associated with similar short-term oncological outcomes, including recurrence-free survival, 12-month recurrence, residual tumor at repeat resection, and detrusor muscle sampling. En bloc resection was associated with selected perioperative advantages, including lower rates of obturator reflex, shorter catheterization, and reduced length of hospitalization, although these findings were accompanied by substantial heterogeneity and were likely influenced, at least in part, by the energy source used. Larger, methodologically standardized trials with longer follow-up are needed to determine whether these procedural advantages translate into durable clinical benefit.

## Declaration of competing interest

The authors declare no conflicts of interest.

## Data Availability

The datasets generated and/or analyzed during the current study are available from the corresponding author upon reasonable request.

## Appendix Supplementary materials

Image, application 1

## Footnotes

Supplementary material associated with this article can be found, in the online version, at doi:10.1016/j.clinsp.2026.101065.
