# Supplementary material for: En bloc versus conventional resection of primary bladder tumor: a systematic review and meta-analysis
Source: Clinics (Sao Paulo). 2026 Jul 23;81:101065. doi: 10.1016/j.clinsp.2026.101065 (PMC13427560; doi:10.1016/j.clinsp.2026.101065)

CLINICS-D-26-00315_Supplementary Material

**Supplementary Table 1** Clinical characteristics of the included studies.

| **Study** | **pTa, n (%)** | | | **pTis, n (%)** | | **pT1, n (%)** | | **pT2, n (%)** | | **Largest diameter (cm), mean (SD)** | | | **Tumoral localization** | | | **Number of lesions, mean (SD)** | | | **Rate of completed ressection, n (%)** | | **Detrusor detection in the specimens, n (%)** | | |
| --- | --- | --- | --- | --- | --- | --- | --- | --- | --- | --- | --- | --- | --- | --- | --- | --- | --- | --- | --- | --- | --- | --- | --- |
|  | **cTURBT** | **ERBT** | **p** | **cTURBT** | **ERBT** | **cTURBT** | **ERBT** | **cTURBT** | **ERBT** | **cTURBT** | **ERBT** | **p** | **cTURBT** | **ERBT** | **p** | **cTURBT** | **ERBT** | **p** | **cTURBT** | **ERBT** | **cTURBT** | **ERBT** | **p** |
| Struck, 2024 | 25 (62.5) | 44 (80) | 0.075 | x | x | 12 (30) | 9 (16.4) | x | x | 2.6 (1.2) | 2.01 (1.13) | x | Left wall 13 (26)  Right wall 20 (40)  Trigonun 7 (14)  Posterior wall 6 (12)  Bladder bottom 0  Bladder neck 2 (4)  Prostate middle lobe 0  Anterior wall 1 (2)  Dome 1 (2) | Left wall 28 (40.6)  Right wall 12 (17.4)  Trigonun 16 (23.2)  Posterior wall 3 (4.4)  Bladder bottom 4 (5.8)  Bladder neck 3 (4.4)  Prostate middle lobe 1 (1.5)  Anterior wall 2 (2.9)  Dome 0 | x | 2 (0) | 2.3 (0.7) | x | x | x | 28 (70) | 37 (67.3) | 0.687 |
| Yuen-Chun Teoh, 2024 | 112 (84) | 110 (77) | x | 3 (2.3) | 2(1.4) | 18 (14) | 31 (22) | x | x | 2.0 (1.0–2.0) | 1.5 (1.0–2.0) | x | Anterior 12 (9.0)  Posterior 20 (15)  Left lateral 35 (26)  Right lateral 34 (26)  Dome 13 (9.8)  Trigone 3 (2.3)  Bladder neck 16 (12) | Anterior 7 (4.9)  Posterior 18 (13)  Left lateral 39 (27)  Right lateral 47 (33)  Dome 16 (11)  Trigone 6 (4.2)  Bladder neck 10 (7.0) | x | Multiple 46 (32) | Multiple 47 (35) | x | x | x | 112 (84) | 119 (83) | 0.8 |
| D'Andrea, 2023 | 146 (63) | 142 (65) | x | 4 (1.7) | 2(0.9) | 57 (25) | 56 (26) | 8 (3.4) | 4 (1.8) | 1.70 (1.00–2.50) | 1.90 (1.00–2.00) | x | Anterior 25 (11)  Posterior 31 (13)  Left 73 (31)  Right 60 (26)  Trigone 44 (19) | Anterior 12 (5.5)  Posterior 33 (15)  Left 76 (35)  Right 58 (26)  Trigone 40 (18) | x | x | x | x | 220 (94) | 211 (96) | 166 (71.1) | 177 (80.7) | 0.01 |
| Badawy, 2023 | 24 (40.0) | 18 (30.0) | 0.498 | x | x | 30 (50.0) | 34 (56.7) | x | x | 1.81±0.58 | 1.85±0.49 | 0.684 | Basal 25 (41.7), Right lateral 8 (13.3), Left lateral 23 (38.4), Basal and right lateral 2 (3.3), Basal and left lateral 2 (3.3) | Basal 18 (30.0), Right lateral 20 (33.3), Left lateral 20 (33.3), Basal and right lateral 2 (3.3), Basal and left lateral 0 | x | Single 55 (91.7), Multiple 5 (8.3) | Single 58 (96.7), Multiple 2 (3.3) | x | 60 (100) | 60 (100) | 44 (73.3) | 57 (95) | 0.001 |
| Gallioli, 2022 | 76 (70) | 90 (64) | 0.9 | 2 (2) | 2 (2) | 15 (14) | 25 (18) | 5 (5) | 6 (4) | < 10 mm 84 (57)  10–30 mm 63 (43) | < 10 mm 92 (47)  10–30 mm 102 (53) | 0.07 | Trigone 24 (16)  Posterior wall 26 (18)  Lateral walls 70 (48)  Anterior wall/dome 18 (12)  Bladder neck 9 (6) | Trigone 24 (12)  Posterior wall 29 (15)  Lateral walls 107 (55)  Anterior wall/dome 23 (12)  Bladder neck 11 (6) | 0.7 | x | x | x | x | x | 137 (93.2) | 180 (92.8) | 0.9 |
| Tripathi, 2021 | x | x | x | x | x | x | x | x | x | 1.74 ±0.62 | 1.71 ±0.64 | x | Lateral wall 10 (19), Posterior wall 12 (23), Postero-lateral wall 18 (35), Posterior wall and trigone 7 (13), Anterior wall only 1 (2), Trigone only 4 (8) | Lateral wall 10 (22), Posterior wall 11 (24), Postero-lateral wall 14 (30), Posterior wall and trigone 5 (11), Anterior wall only 1 (2), Trigone only 5 (11) | x | Single 36 (83.7), Multiple 7 (16.3) | Single 35 (87.5), Multiple 5 (12.5) | x | x | x | 24 (55.8) | 22 (55) | 0.884 |
| Miyake, 2021 | x | x | x | x | x | x | x | x | x | x | x | x | x | x | x | x | x | x | x | x | x | x | x |
| Razzaghi, 2021 | 26 (66.7) | 25 (62.5) | x | 0 | 0 | 13 (33.3) | 15 (37.5) | x | x | 2.22 (0.81) | 1.98 (1.07) | x | Lateral 15 (38.5), Other 24 (61.5) | Lateral 18 (45), Other 22 (55) | x | Single 23 (58.9), Multiple 16 (41.1) | Single 25 (62.5), Multiple 15 (37.5) | x | x | x | x | x | x |
| Alkhaledi, 2021 | x | x | x | x | x | x | x | x | x | x | x | x | x | x | x | x | x | x | 23 (88.5) | 26 (100) | x | x | x |
| Fan, 2021 | 104 (88.9) | 91 (78.5) | x | x | x | 13 (11.1) | 25 (21.5) | x | x | 1.5 (1–2) | 1.5 (1.2–1.5) | x | Lateral wall 47 (40.2), Anterior 3 (2.6), Posterior 13 (11.1), Trigone area 49 (41.9), Neck 5 (4.3) | Lateral wall 61 (52.6), Anterior 4 (3.4), Posterior 13 (11.2), Trigone area 36 (31.0), Neck 2 (1.7) | 0.261 | 1 (1–1) | 1 (1–1.7) | x | x | x | 84 (71.8) | 104 (89.7) | 0.005 |
| Hashem, 2021 | 3 (6) | 2 (4) | x | 46 (93) | 42 (84) | x | x | x | x | 2.9 (1.4) | 3.2 (1.1) | x | Posterior 13 (26.0), Lateral 13 (26.0), Dome 2 (4.0), Multiple 22 (44.0) | Posterior 11 (22.0), Lateral 18 (36.0), Dome 4 (8.0), Multiple 17 (34.0) | 0.52 | Single (56), Multiple (44) | Single (66), Multiple (34) | x | 13 (26.5) | 3 (7) | 31 (62) | 49 (98) | <0.001 |
| Gakis, 2020 | 42 (71.2) | 50 (89.3) | x | 0 | 0 | 17 (28.8) | 6/56 (10.7) | x | x | x | x | x | x | x | x | x | x | x | 5 (8.5) | 30 (53.6) | 36 (66.7) | 41 (77.4) |  |
| Balan, 2018 | 23 (51.1) | 24 (53.3) | x | x | x | 22 (48.9) | 21 (46.7) | x | x | 1.69 | 1.82 | x | x | x | x | x | x | x | x | x | x | x | x |
| Huang, 2016 | 35 (50.0) | Thulium: 40 (57.1)  Holmium: 37 (52.9) | > 0.05 | 8 (11.4) | Thulium: 7 (10.0)  Holmium: 5 (7.1) | 27 (38.6) | Thulium: 23 (32.9)  Holmium: 28 (40.0) | x | x | 1.53 (0.20) | Thulium 1.63 (0.32)  Holmium 1.58 (0.51) | > 0.05 | Lateral 25 (35.7)  Other 45 (64.3) | Thulium:  Lateral 23 (32.9)  Other 47 (67.1)  Holmium:  Lateral 28 (40.0)  Other 42 (60.0) | x | 2.53 (1.21) | Thulium: 2.74 (1.52)  Holmium: 2.43 (1.33) | >0.05 | x | x | x | x | x |
| Zhang, 2015 | 107 (74.8) | 106 (71.1) | x | x | x | 36 (25.2) | 43 (28.9) | x | x | < 3cm 95 (66.4)  ≥ 5.8cm 48 (33.6) | < 3cm 98 (65.8)  ≥ 5.8cm 51 (34.2) | x | x | x | x | Single 78 (54.5); Multiple 65 (45.5) | Single 77 (51.7); Multiple 72 (48.3) | x | 126/143 (88.1) | 122/149 (81.9) | 134/143 (93.7) | 131/149 (87.9) | x |
| Chen, 2015 | 55 (77.5) | 43 (60.6) | x | 1 (1.4) | 3 (4.2) | 15 (21.1) | 25 (35.2) | x | x | 2.3 (1.2) | 2.6 (1.4) | 0.071 | Anterior 17 (15.3); Posterior 18 (7.2); Right lateral 29 (26.1); Left lateral 34 (30.6); Triangle area 15 (13.5); Bladder neck 8 (7.2) | Anterior 10 (7.8); Posterior 17 (13.3); Right lateral 28 (21.9); Left lateral 45 (35.2); Triangle area 13 (10.2); Bladder neck 15 (11.7) | x | 1.7 (1.7) | 1.8 (1.5) | x | 100 | 100 | x | x | x |
| Liu, 2013 | 34 (60.7) | 37 (57.8) | x | x | x | 22 (39.3) | 27 (42.2) | x | x | 1.28 (0.31) | 1.31 (0.23) | x | Lateral 21 (37.5); Other 35 (62.5) | Lateral 24 (37.5); Other 40 (62.5) | x | 2.7 (1.5) | 2.8 (1.2) | x | 56 (100) | 64 (100) | x | x | x |

**Supplementary Table 2** Surgery and Instillation treatment characteristics of the included studies.

| **Study** | **Source Energy** | **Visualization method, n (%)** | | **Instillation treatment, n (%)** | |
| --- | --- | --- | --- | --- | --- |
|  |  | **cTURBT** | **ERBT** | **cTURBT** | **ERBT** |
| Struck, 2024 | Bipolar, monopolar, Holmium laser | White light 25 (62.5), NBI 8 (20.0), HEXVIX 4 (10.0), SPIES 2 (5.0), HEXVIX 1 (2.5) | White light 34 (59.6), NBI 20 (35.1), HEXVIX 2 (3.5), SPIES 0 (0), HEXVIX 1 (1.8) | x | x |
| Yuen-Chun Teoh, 2024 | Bipolar | White light | White light | Mitomycin 102 (77) | Mitomycin 106 (74) |
| D'Andrea, 2023 | Bipolar, laser, monopolar | IMAGE1 S 21 (9.0), Narrowband 122 (52), Protodynamic 90 (39) | IMAGE1 S 26 (12), Narrowband 100 (46), Protodynamic 93 (42) | An immediate instillation of postoperative chemotherapy was administered within 24h of TURB in the absence of contraindications. | |
| Badawy, 2023 | Thulium laser, monopolar | x | x | Doxorubicin (50 mg) within 6 h postoperatively. | |
| Gallioli, 2022 | Bipolar, monopolar, Thulium laser | x | x | One-shot instillation of mitomycin C (40 mg) or epirubicin (50 mg). | |
| Tripathi, 2021 | KTP laser, bipolar | x | x | Mitomycin-C (40 mg) intravesically. | |
| Miyake, 2021 | Standard loop eletrode | x | x | Epirubicin (60mg) within 24h postoperatively. | |
| Razzaghi, 2021 | Holmium laser, monopolar | x | x | x | x |
| Alkhaledi, 2021 | Holmium laser, monopolar | x | x | x | x |
| Fan, 2021 | Green-light | x | x | Intravesical pirarubicin (30 mg/50 mL) once within 24h, then once a week for 8-weeks, and once a month for a total of 1-year | x |
| Hashem, 2021 | Holmium laser | x | x | Epirubicin 1 (1.8), BCG 45 (91.8) | Epirubicin 4 (9.4), BCG 39 (88.6) |
| Gakis, 2020 | HybridKnife^®^, Water-jet unit (ErbeJet^®^ 2), Loop resectoscope | White light, Blue light | White light, Blue light | MMC 14 (23.7); BCG 7 (11.9); MMC and/or BCG 20 (33.9) | MMC 7 (12.5); BCG 6 (10.7); MMC and/or BCG 13 (23.2) |
| Balan, 2018 | Bipolar plasma vaporization | x | x | x | x |
| Huang, 2016 | Thulium (RevoLix 2-micron) laser, Holmium laser | x | x | Epirubicin (40 mg/40 mL) 1-week after the operation, weekly for 8-weeks. Followed by monthly to 12 months. | |
| Zhang, 2015 | Thulium laser | x | x | Epirubicin (40 mg) once a week for 8-weeks. | |
| Chen, 2015 | Thulium laser | x | x | Epirubicin (50 mg/50mL) weekly for 8-weeks. Followed by monthly to 12-months. | |
| Liu, 2013 | Thulium laser, monopolar | x | x | Epirubicin (40 mg) weekly for 8-weeks, beginning 1-week after the operation. Followed with monthly for 12-months. | |

**Supplementary Figure 1** Study risk of Bias.


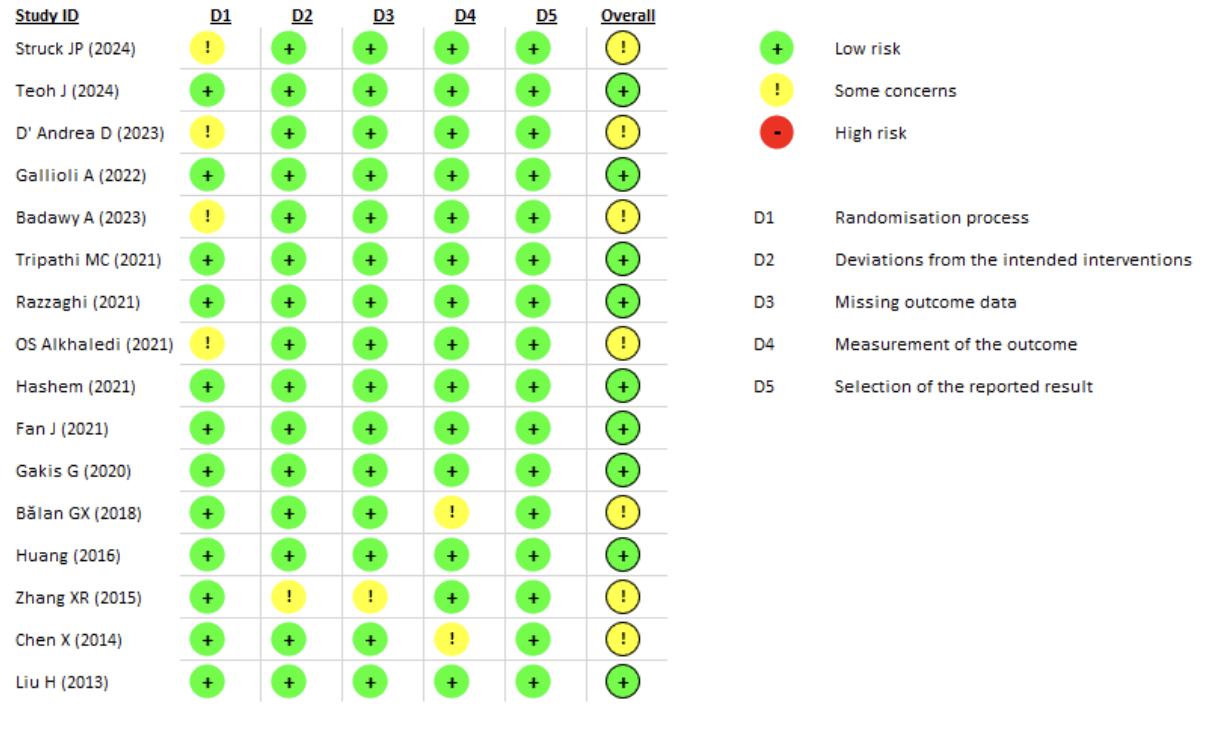


**Supplementary Figure 2** Funnel plot for publication bias.


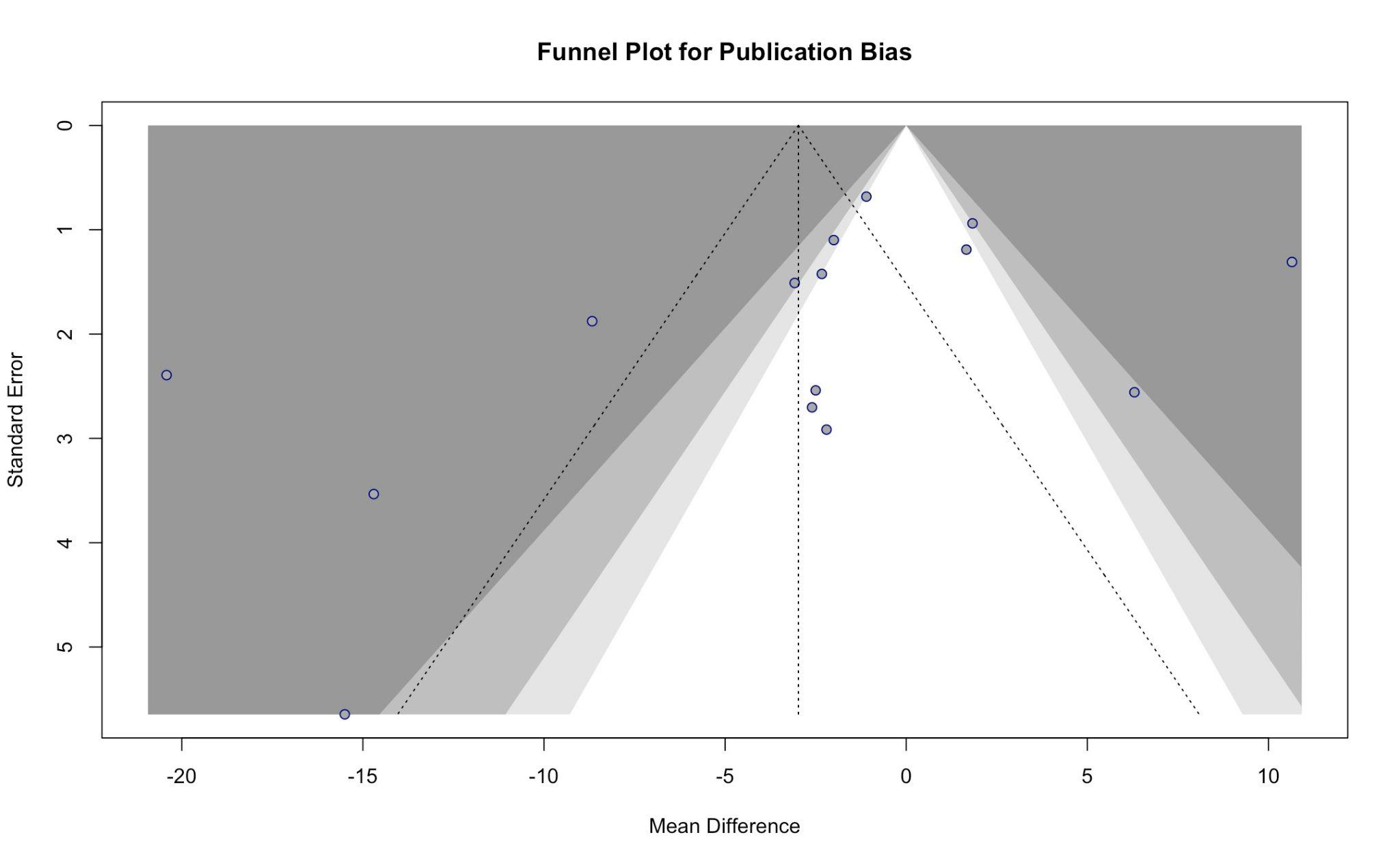

Supplement: Supplementary file 1 [file mmc1.docx]
